# Development and performance evaluation of a deep learning lung nodule detection system

**DOI:** 10.1186/s12880-022-00938-8

**Published:** 2022-11-22

**Authors:** Shichiro Katase, Akimichi Ichinose, Mahiro Hayashi, Masanaka Watanabe, Kinka Chin, Yuhei Takeshita, Hisae Shiga, Hidekatsu Tateishi, Shiro Onozawa, Yuya Shirakawa, Koji Yamashita, Jun Shudo, Keigo Nakamura, Akihito Nakanishi, Kazunori Kuroki, Kenichi Yokoyama

**Affiliations:** 1 Department of Radiology, Faculty of Medicine, Kyorin University, 6-20-2, Shinkawa, Mitaka-shi, Tokyo, Japan; 2 Imaging Technology Center, ICT Strategy Division, Fujifilm Corporation, 2-26-30, Nishi-Azabu, Minato-ku, Tokyo, Japan; 3 Department of Radiology, Kyorin University Hospital, 6-20-2, Shinkawa, Mitaka-shi, Tokyo, Japan

**Keywords:** Artificial intelligence, Lung nodule, Computer aided detection, Deep learning

## Abstract

**Background:**

Lung cancer is the leading cause of cancer-related deaths throughout the world. Chest computed tomography (CT) is now widely used in the screening and diagnosis of lung cancer due to its effectiveness. Radiologists must identify each small nodule shadow from 3D volume images, which is very burdensome and often results in missed nodules. To address these challenges, we developed a computer-aided detection (CAD) system that automatically detects lung nodules in CT images.

**Methods:**

A total of 1997 chest CT scans were collected for algorithm development. The algorithm was designed using deep learning technology. In addition to evaluating detection performance on various public datasets, its robustness to changes in radiation dose was assessed by a phantom study. To investigate the clinical usefulness of the CAD system, a reader study was conducted with 10 doctors, including inexperienced and expert readers. This study investigated whether the use of the CAD as a second reader could prevent nodular lesions in lungs that require follow-up examinations from being overlooked. Analysis was performed using the Jackknife Free-Response Receiver-Operating Characteristic (JAFROC).

**Results:**

The CAD system achieved sensitivity of 0.98/0.96 at 3.1/7.25 false positives per case on two public datasets. Sensitivity did not change within the range of practical doses for a study using a phantom. A second reader study showed that the use of this system significantly improved the detection ability of nodules that could be picked up clinically (p = 0.026).

**Conclusions:**

We developed a deep learning-based CAD system that is robust to imaging conditions. Using this system as a second reader increased detection performance.

**Supplementary Information:**

The online version contains supplementary material available at 10.1186/s12880-022-00938-8.

## Background

Lung cancer is one of the leading causes of cancer-related deaths, with about 1.8 million deaths in 2020 [1]. Computed tomography (CT) is indispensable for the early detection and diagnosis of lung cancer [2–5]. Identifying comparatively small abnormalities such as nodules is a laborious task, and small lesions, particularly sub-solid ground-glass nodules, may be overlooked. Due to the advent of multi-row detector CT and other advances in imaging technology, the volume of imaging data obtained from each single study is increasing annually. Combined with the shortage of radiologists [6, 7], the burden on radiologists continues to increase. The development of a computer-aided detection (CAD) system for identifying nodules is expected to assist radiologists by preventing lung cancers being overlooked.

Artificial intelligence (AI) has been shown to provide high performance in a variety of image recognition tasks [8, 9]. Many technologies have been proposed to assist in the detection and management of pulmonary nodules [10], especially since the advent of deep learning [11–15]. For reliable nodule management, such an AI system needs to be robust in various imaging conditions as well as clinically useful. Regarding robustness to imaging conditions, randomized, controlled trials have recently shown the effectiveness of CT screening for lung cancer [2, 3], and AI is expected to provide stable performance for low-dose images with high noise levels. Under these circumstances, several studies have been conducted to evaluate nodule detection in low-dose images [16]. However, only a few studies have evaluated the relationship between noise level and AI detection performance using phantoms [17]. The clinical usefulness of nodule detection systems has been evaluated in several studies [18–20]. Liu et al. reported that their CAD potentially enhanced the manual identification of pulmonary nodules and reduced reading time when used for assistance [21]. Although there may be differences in clinical usefulness depending on reading experience, no studies to date have adequately investigated clinical usefulness.

In the present study, a lung nodule CAD system was developed based on a 3D convolutional neural network (CNN). A chest phantom was used to create images at varying radiation doses, and the robustness of the system with respect to differences in radiation dose was evaluated. In addition, ten doctors with varying levels of experience in chest imaging diagnosis were asked to interpret scans with and without this system and evaluate its clinical usefulness (i.e., whether it enables the reader to identify clinically significant lesions without omission when used as a second reader tool).

## Methods

### Clinical data

For the purpose of algorithm development, 1177 chest CT scans scanned at Kyorin University Hospital between April 2013 and March 2018 that showed at least one lung nodule were retrospectively collected. Cases with diffuse lung disease across a wide area or with numerous nodules (> 20 nodules) were excluded. To reduce the institutional dependency of the algorithm, public data (LIDC-IDRI [22]) and patients scanned at a single Japanese institution were also added. After patients with numerous nodules (> 20 nodules) and surgical patients were excluded, 2027 scans of 1799 patients were used to develop the algorithm (30 of them were used as validation data for parameter determination). The LIDC-IDRI dataset contains 1018 scans that have been made public, but after excluding patients with no nodules or numerous nodules and those with diffuse lung disease across a wide area, 127 of the resulting 953 scans were used for the internal validation dataset, and the remaining 826 were used for algorithm training.

In addition to the above data, the following two datasets were used as validation datasets for the assessment of detection performance.

1. SPIE-AAPM Lung CT Challenge [23]: The SPIE-AAPM dataset is the data for the Grand Challenge in Lung Nodule Classification set by the International Society for Optics and Photonics (SPIE) in collaboration with the American Association of Physicists in Medicine (AAPM) and the National Cancer Institute. It comprises 70 cases with annotated nodules that were published as training data.
2. LNDb [24]: The LNDb dataset is the data for the Nodule Detection, Segmentation Texture Characterization, and Fleischner Classification Grand Challenge published by the INESC TEC in Portugal. It comprises 294 annotated cases published from among chest CTs scanned between 2016 and 2018 at the Centro Hospitalar e Universitário de São João in Porto, Portugal.

### Ground truth data creation

The positions of the lung nodules in the 1997 scans in the algorithm training dataset were annotated by non-experts who received training on how to annotate lung nodules, and all data were checked by a board-certified radiologist with 22 years of experience in image interpretation. Lung nodules were annotated if their major axis diameter measured ≥ 3 mm for solid or part-solid nodules and ≥ 5 mm for ground-glass nodules. Information on the annotated lung nodules is given in Table 1.

**Table 1:** Details of training data

Characteristic	#
Data information	
No. of chest CTs	1997
No. of patients	1769
No. of men*	688
No. of women*	397
Mean age* (y)	67.7 ± 10.9
Slice thickness (mm)	
≤ 1.0	475
≤ 2.0	547
≤ 3.0	308
≤ 4.0	1
≤ 5.0	437
Contrast	
Contrast-enhanced	386
Non-contrast-enhanced	1611
Kernel type	
Lung kernel	1268
Abdomen kernel	533
Bone kernel	196
Manufacturer	
Toshiba Medical Systems Corp	1001
GE Healthcare	511
Siemens Healthineers AG	423
Philips	62
Nodule information	
No. of nodules	10,467 (5.2/scan)
Mean nodule size (mm)	8.3 ± 7.9
No. of nodules according to size (mm)	
≤ 5.0	4150
≤ 10.0	4406
≤ 20.0	1071
≤ 30.0	538
≤ 40.0	156
≤ 50.0	91
> 50.0	55
Lobular distribution	
Right upper	2852
Right middle	980
Right lower	2177
Left upper	2601
Left lower	1857

^*^Sex/Age data have been removed from some of the LIDC-IDRI data, so that they are not included in the count

### Development of lung nodule CAD

**Fig. 1:**
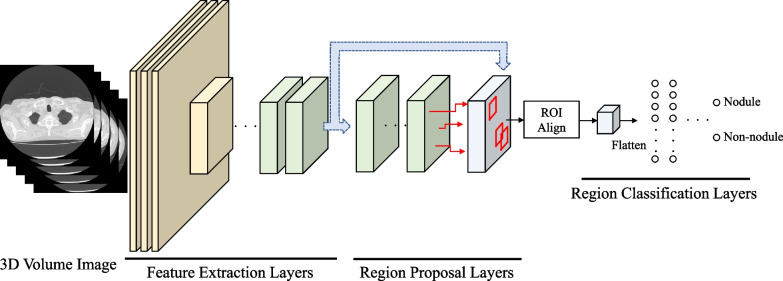
Overview of the detection network. Feature extraction layers extract characteristics from 3D image data by 3D convolution, and region proposal layers output multiple candidate regions. Region classification layers determine whether each candidate region is a nodule, and make this the final output

Based on the Faster R-CNN [25], a detection AI with a Region Proposal Network consisting of 27 convolutional layers, each followed by a batch normalization layer and an activation layer was designed (Fig. 1). Distinguishing blood vessels and nodules from two-dimensional axial slices is very difficult. Therefore, it was decided to use 3D convolution layers that can extract 3D information.

For the input image, the raw pixel value of the DICOM image was corrected based on the values of the rescale intercept and rescale slope, and normalized to 0–1 within the range − 1500 to 300. As preprocessing, the image spacing was rescaled to 1 mm. Images were processed by the lung-field extraction AI installed in the SYNAPSE SAI viewer (Fujifilm Corporation, Tokyo, Japan). A circumscribed rectangle was calculated for the extracted lung field region, and the region within the rectangle was cropped out and input into the network. To remove false positives (FPs) from outside the lung fields from the detection results, post-processing using the lung field extraction results was conducted to determine whether each candidate detected by the CAD system was located within the lung fields. Changes in rotation, scale, and sharpness and Gaussian noise addition were carried out as data augmentation during training (see Table 2 for more details).

**Table 2:** Details of data augmentation during training

Data augmentation type	Method
Rotation	Randomly rotate around the z-axis in the range of − 10 to 10 degrees
Scale	Randomly change the size by − 15 to 15% in each axis direction
Sharpness	Change the sharpness according to the following formula
	$$I_{out} = I_{in} + \alpha \left( {I_{in} - f\left( {I_{in} } \right)} \right)$$
	*I*_*in*_: input image; *I*_*out*_: output image; *f*(*I*_in_): image processed with a Gaussian filter with a standard deviation of 3.0; α: parameter (randomly selected within the range of 0.0–5.0
Smoothing	Process with a Gaussian filter that randomly sets the standard deviation in the range of 0.5–2.0
Gaussian Noise	Add Gaussian noise generated in the range of standard deviation 0.0–0.2

The coordinates of the nodule candidates detected and the confidence level of each candidate (on a continuous scale from 0–1) were output by the network. A threshold of 0.56 was set, which is the value for 2.0 FPs/case in the validation dataset, and the detection results for candidates that were output with a confidence level exceeding this threshold were displayed.

### Validation of CAD performance

To evaluate the nodule detection performance of the CAD, internal and external validation tests were conducted. The positions of the nodules are all annotated in the LIDC-IDRI, SPIE-AAPM, and LNDb datasets. Following the evaluation methods used in the LUNA16 and LNDb challenges, respectively, the ground truth for the LIDC-IDRI dataset was solely nodules detected by three of four annotating radiologists, whereas that for the LNDb dataset was solely nodules detected by two of five annotating radiologists. Details on each validation dataset are given in Additional file 4: Table S1. The evaluation metrics used comprised the sensitivity and number of FPs per scan, as well as FROC analysis [26] and the competition performance metric (CPM) [27]. The CPM was the mean sensitivity at threshold FP rates of 1/8, 1/4, 1/2, 1, 2, 4, and 8 per scan.

### Evaluation of robustness to changes in radiation dose

To confirm that the detection performance of the CAD system was stable and independent of the radiation dose during scanning, a chest phantom (N-1 LUNGMAN, Kyoto Kagaku Corporation, Kyoto, Japan) fitted with multiple simulated nodules at different doses was scanned, detection processing was carried out on the resulting images, and the results were confirmed.

All CT scans were acquired with a 320-row CT scanner (Aquilion ONE, Canon Medical Systems Corporation, Otawara, Japan). The scanning parameters were as follows: tube voltage 120 kVp and image noise standard deviation (SD) 10, 15, 20, and 25. These parameters were set in accordance with the Japanese guidelines for X-ray CT scanning [28]. These guidelines state that, for chest imaging diagnosis, the SD should be set within the range 10–12, and for lung cancer screening, it should be set within the range 20–25. Scanning was performed using CT-auto exposure control, and the iterative approximation method of reconstruction was carried out with a slice thickness of 2.0 mm.

The following simulated nodules were prepared for implantation in the chest phantom.

1. Types: Pure ground-glass nodules (pure GGNs) and solid nodules: − 630 Hounsfield Units (HU) simulated nodules were used as pure GGNs, and 100 HU simulated nodules were used as solid nodules.
2. Sizes: Simulated solid nodules with major axis diameters of 3, 5, and 8 mm and simulated GGNs with major axis diameters of 5 and 8 mm were used.

These five simulated nodules were placed randomly within the lung fields and scanned using each of the scanning parameters described above. This process was repeated five times. The sensitivity and number of FPs per image and FROC analysis were used as evaluation metrics, and whether there were any differences between scanning parameters was investigated.

### Reader performance test

**Fig. 2:**
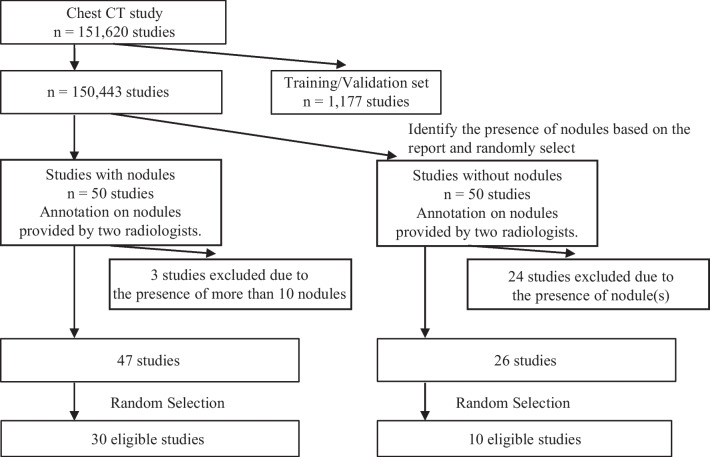
Scan selection flowchart for the reader performance test. From the chest CTs scanned in our hospital that were not used for algorithm training, 50 in which nodules were mentioned in the radiologist’s report and 50 in which no nodule was mentioned were randomly selected retrospectively. These scans were examined by two board-certified radiologists who identified nodules. Three of these scans were excluded because > 10 nodules were identified, after which 30 images with nodules and 10 without nodules were randomly selected and used for the reader performance test

Whether using the CAD as a second reader could assist doctors and reduce the number of nodules that are missed was investigated using internal data. Figure 2 shows the selection protocol for the images evaluated. Two board-certified radiologists, who had 22 and 17 years, respectively, of experience with chest image interpretation examined the images and identified nodules. To prevent omission of nodules, the nodules annotated by at least one doctor were designated as ground truth. The nodules were defined as “nodules requiring follow-up” according to the following two criteria.

Criterion 1: All nodules in patients with a history of malignant neoplasm or confirmed lung cancer, or those with a mass/nodule strongly suspected to be lung cancer.

Criterion 2: Nodules of major axis diameter ≥ 6 mm (based on the Fleischner Study [4]).

The breakdown of the ground truth for each of the criteria is provided in Table 3.

**Table 3:** Details of data used for the reader performance test

Characteristic	#	
	Criterion 1	Criterion 2
Data information		
No. of chest CTs	40	
No. of positive cases	17	29
No. of negative cases	23	11
No. of patients	40	
No. of men	29	
No. of women	11	
Mean age* (y)	64.7 ± 13.6	
Slice thickness (mm)		
2.0	40	
Contrast		
Non contrast	40	
Kernel type		
Lung kernel	40	
Manufacturer		
Toshiba Medical Systems Corp	40	
Nodule information		
No. of total nodules	115	
No. of nodules (follow-up required)	57 (3.4/scan)	53 (1.8/scan)
No. of nodules according to size (mm)		
≤ 10.0	38	28
≤ 20.0	11	16
≤ 30.0	3	4
> 30.0	5	5
Lobular distribution		
Right upper	11	14
Right middle	7	4
Right lower	17	16
Left upper	9	8
Left lower	13	11
Internal characteristics		
Solid	43	34
Part solid	8	12
GGN	6	7

Ten doctors with different levels of experience took part in the study as readers. These readers were divided into three groups by their level of experience in chest imaging diagnosis (Group 1: ≥ 7 years of experience, n = 3; Group 2: 2–6 years of experience, n = 4; and Group 3: < 2 years of experience, n = 3). Each reader first conducted a search for lung nodules without using the CAD system for 40 cases. When they identified a lung nodule, they annotated the lesion on the CT image and scored it on a free scale according to whether they considered it required follow-up. After this, the CAD output was overlaid on the image as a bounding box, and the readers checked it before repeating the task. Three test cases were prepared to accustom them to the procedure. Considering the time available for image interpretation in actual clinical practice, the readers were requested to take less than 5 min to check each image with and without CAD (total 10 min). For statistical analysis, figures of merit were calculated using Jackknife Free-Response Receiver Operation Characteristic (JAFROC) analysis [29], with *p* < 0.05 considered to indicate significance.

## Results

### Validation of CAD performance

**Fig. 3:**
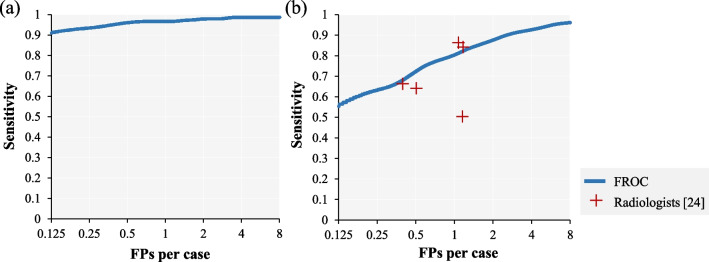
Internal and external validation results. **a** FROC curve for the LIDC-IDRI dataset. **b** FROC curve for the LNDb dataset. The red markers show the detection performance of the five radiologists [24]

The results of internal and external dataset evaluations are shown in Table 4 and Fig. 3a, b. There was no great difference in sensitivity between the internal and external datasets. The present results provided higher sensitivity compared to the latest study [14, 30] evaluating performance using the SPIE-AAPM dataset (0.964@8.0 FPs). However, the present evaluation of the LNDb dataset produced a much greater number of FPs compared with the evaluation of the LIDC-IDRI dataset, and the CPM score was also worse. The ground truth for the LNDb dataset was prepared by five radiologists (with ≥ 4 years of experience), and it was confirmed that the performance of the present CAD system was almost equivalent to the lung nodule detection performance [24] of those radiologists.

**Table 4:** Validation results for each dataset

Dataset	No.of Nodules	Sensitivity				FPs per scan (FPs in total)	CPM score
		Total	Solid	Part-solid	GGN		
*Internal*							
LIDC-IDRI (127 cases)	152	0.980 (149/152)	0.985 (134/136)	1.000 (7/7)	0.889 (8/9)	3.10 (394)	0.960
*External*							
SPIE-AAAM (70 cases)	83	0.988 (82/83)	0.984 (63/64)	1.000 (15/15)	1.000 (4/4)	5.88 (352)	0.893
LNDb (177 cases)	221	0.959 (212/221)	0.970 (193/199)	1.000 (7/7)	0.800 (12/15)	7.25 (1284)	0.783

Additional file 1: Fig. S1 shows examples of true positives (TPs), FPs, and false negatives (FNs). Many different types of nodules were detected, including small solid nodules and well-demarcated ground-glass nodules. Most of the FPs were pleural inflammation or peripheral vessels, and some of the inflammatory shadows were picked up with a low confidence level. Most of the FNs were faint, poorly demarcated ground-glass nodules, and nodules with a rare shape or adjacent to the diaphragm (details are shown in Additional file 4: Table S2).

### Evaluation of robustness to changes in radiation dose

Sensitivity did not vary at different SD values, being 96.0% for SDs of 10, 15, 20, and 25. The number of FPs stayed within the range of 0.4–0.8 per scan (Table 5). At SD 20, the CPM score decreased, but this was because one of the five scans had a false positive with a confidence level close to that of the simulated nodule, and it was only at SD 20 that a false positive had a confidence level greater than that of a simulated nodule. Except for this scan, there was no change in the detection rates for all other scans, and the variability of the CPM score was also kept below 1%.

**Table 5:** Detection results for each SD value

SD	Sensitivity	FPs per scan (no. of FPs)	CPM score
10	0.96 (24/25)	0.6 (3)	0.951
15	0.96 (24/25)	0.4 (2)	0.950
20	0.96 (24/25)	0.8 (4)	0.941
25	0.96 (24/25)	0.8 (4)	0.950

### Reader performance test

The detection rate of the CAD system for the 115 nodules in the 40 scans was 89.6%. The detection rate for nodules included in the ground truth data was 89.5% according to Criterion 1 and 98.1% according to Criterion 2. The false-positive rate was 0.63 per scan. The dataset included five cases with no nodules at all, and the specificity for these cases was 0.8.

**Fig. 4:**
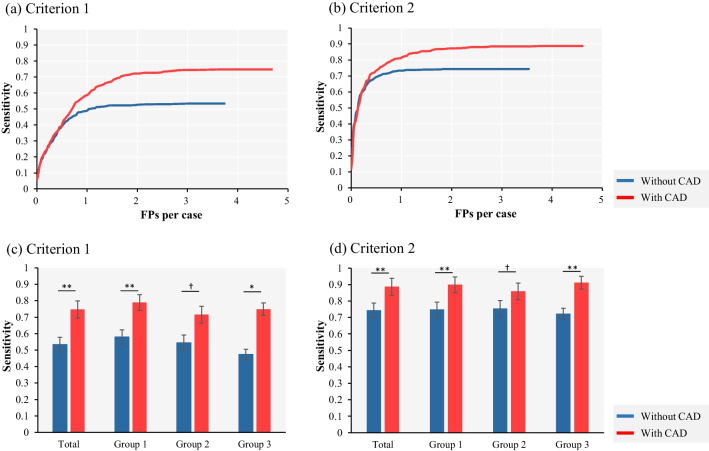
Reader performance test results. **a** and **b** Mean FROC curves for readers using criterion 1 and 2. The red lines show the FROC curves with CAD use, and the blue lines without CAD use. **c** and **d** Detection sensitivities with and without CAD use using criteria 1 and 2. The red bars show the sensitivity with CAD use and the blue bars without CAD use. **Significant difference (*p* < 0.01). *Significant difference (*p* < 0.05). ^†^Significant tendency (*p* < 0.10; two-tailed paired *t*-test)

JAFROC random case and random reader analysis showed that the figure-of-merit was significantly increased when using the CAD system as a second reader tool (Criterion 1: *p* = 0.026; Criterion 2: *p* = 0.012; Fig. 4a, b). The detection sensitivity improved for all reader groups, and a two-tailed paired *t*-test showed that the improvements were significant for Group 1 and Group 3 (Fig. 4c, d). The FROC curves for each group are shown in Additional file 2: Fig. S2. The number of nodules identified per reader was 2.19 per scan before CAD use and 3.13 per scan after CAD use. The mean interpretation time per case is shown in Table 6. In this experiment, which used CAD as a 2nd reader tool, it took approximately 1 min to review the CAD results. However, in Group 3, which had little experience in chest imaging diagnosis, review took approximately twice as long as the other groups.

**Table 6:** Mean interpretation time per case (mean ± SD)

Nodule type	Total	Group 1	Group 2	Group 3
Total	5:22 ± 3:49	3:33 ± 2:29	5:27 ± 4:34	7:04 ± 4:09
Without CAD	4:09 ± 2:36	2:35 ± 1:32	4:31 ± 3:36	5:13 ± 2:19
CAD result review	1:13 ± 1:13	0:58 ± 0:57	0:56 ± 0:57	1:52 ± 1:50

Table 7 shows an analysis of the effect of CAD use by nodule characteristics, size, and location. In terms of nodule types, the detection rate of GGNs was more greatly improved by CAD use than were those of solid and part-solid nodules, even though the model still struggled with GGNs relative to the other types of nodules. This tendency was particularly marked in Group 3, and by using CAD, the sensitivity for GGNs improved to almost the same level as Group 1. In terms of size, CAD use improved the detection rate of nodules of major axis diameter ≤ 1 cm, but there was almost no increase in sensitivity for nodules measuring ≥ 2 cm for all reader groups. In terms of nodule location, CAD use greatly improved the detection rate of nodules attached to the interlobular fissures. In addition, the detection rate of nodules located in lower lobes in Group 3 was lower than that in Group 1, but it improved to the same extent as Group 1 with CAD. Additional file 3: Fig. S3 shows examples of the nodules detected by the CAD system. The system was clearly effective for nodules that are difficult to identify, such as solid nodules adjacent to blood vessels.

**Table 7:** Changes in detection sensitivity for nodules of different types, sizes, and positions (mean ± SD)

	N	Without CAD				With CAD			
		Total	Group 1	Group 2	Group 3	Total	Group 1	Group 2	Group 3
*Nodule type*									
Solid	43	0.59 ± 0.07	0.64 ± 0.03	0.59 ± 0.06	0.54 ± 0.08	0.81 ± 0.09	0.85 ± 0.02	0.78 ± 0.12	0.80 ± 0.08
Part solid	8	0.56 ± 0.16	0.58 ± 0.16	0.63 ± 0.18	0.46 ± 0.06	0.71 ± 0.12	0.75 ± 0.06	0.66 ± 0.05	0.75 ± 0.06
Pure GGN	6	0.08 ± 0.11	0.17 ± 0.14	0.08 ± 0.08	0.00 ± 0.00	0.35 ± 0.17	0.39 ± 0.08	0.29 ± 0.18	0.39 ± 0.16
*Nodule size (mm)*									
≤ 10	38	0.37 ± 0.08	0.41 ± 0.03	0.39 ± 0.08	0.31 ± 0.05	0.66 ± 0.11	0.71 ± 0.04	0.63 ± 0.13	0.66 ± 0.09
≤ 20	11	0.78 ± 0.12	0.85 ± 0.11	0.80 ± 0.08	0.70 ± 0.11	0.88 ± 0.09	0.91 ± 0.04	0.86 ± 0.08	0.88 ± 0.07
≤ 30	3	1.00 ± 0.00	1.00 ± 0.00	1.00 ± 0.00	1.00 ± 0.00	1.00 ± 0.00	1.00 ± 0.00	1.00 ± 0.00	1.00 ± 0.00
> 30	5	0.92 ± 0.10	1.00 ± 0.00	0.85 ± 0.09	0.93 ± 0.09	0.96 ± 0.08	1.00 ± 0.00	0.90 ± 0.09	1.00 ± 0.09
*Nodule position*									
Right upper lobe	11	0.64 ± 0.10	0.61 ± 0.04	0.68 ± 0.10	0.61 ± 0.11	0.86 ± 0.13	0.85 ± 0.09	0.86 ± 0.13	0.88 ± 0.15
Right middle lobe	7	0.24 ± 0.11	0.24 ± 0.07	0.25 ± 0.16	0.24 ± 0.07	0.54 ± 0.13	0.57 ± 0.07	0.54 ± 0.17	0.52 ± 0.12
Right lower lobe	17	0.54 ± 0.08	0.61 ± 0.06	0.56 ± 0.03	0.43 ± 0.03	0.69 ± 0.11	0.73 ± 0.08	0.66 ± 0.06	0.71 ± 0.07
Left upper lobe	9	0.48 ± 0.12	0.56 ± 0.09	0.44 ± 0.14	0.44 ± 0.09	0.71 ± 0.15	0.85 ± 0.21	0.64 ± 0.12	0.67 ± 0.09
Left lower lobe	13	0.64 ± 0.10	0.72 ± 0.04	0.63 ± 0.10	0.56 ± 0.10	0.85 ± 0.11	0.90 ± 0.10	0.81 ± 0.11	0.87 ± 0.06
Attached to parietal pleura	16	0.71 ± 0.11	0.77 ± 0.13	0.69 ± 0.12	0.69 ± 0.05	0.78 ± 0.07	0.83 ± 0.05	0.73 ± 0.08	0.79 ± 0.03
Attached to visceral pleura	7	0.74 ± 0.09	0.76 ± 0.07	0.71 ± 0.00	0.76 ± 0.13	0.84 ± 0.11	0.86 ± 0.07	0.82 ± 0.12	0.86 ± 0.13
Attached to interlobular fissure	11	0.43 ± 0.09	0.42 ± 0.04	0.43 ± 0.13	0.42 ± 0.04	0.68 ± 0.10	0.67 ± 0.04	0.66 ± 0.14	0.73 ± 0.04

## Discussion

A lung nodule detection system was developed using deep learning that is both accurate and robust to radiation dose. Using this system as a second reader significantly decreased the number of missed nodules that required follow-up.

One strength of this study is that a large training dataset of high quality was constructed. This training dataset consisted of data from multiple institutions, and it was annotated under the supervision of radiologists. Compared with the datasets used in previous studies [16], the present dataset had more nodules per scan (5.2) and contained comprehensive annotations including even small nodules that are at risk of being overlooked and cost a lot to create ground truth. Training with these data might have made this system capable of stable detection irrespective of the radiation dose, scanning modality, or type of nodule.

In comparison with previous studies, the present study has two achievements. The first is that, in phantom experiments, the stability of the CAD system in detecting nodules irrespective of image noise level due to differences in radiation dose was demonstrated quantitatively. Deep learning-based AI systems generally exhibit poor robustness to subtle changes in images [31, 32]. Because image quality varies as a result of differences in radiation dose, there are concerns that it may also affect detection results. Liu et al. [21] investigated radiation doses in retrospectively collected data and evaluated the effect of differences in dose on detection performance. However, their method is not capable of assessing the pure effect of image noise level due to differences in dose alone. In the present phantom experiments, it was possible to evaluate the effect of differences in dose on detection performance, independently of the effects of individual differences between subjects and differences between devices. It was found that, although changes in the SD value did slightly affect the detection results, there were almost no changes in sensitivity or detection performance. This may have been because robustness to slight changes in images had been achieved by data augmentation in the form of changes in sharpness and the addition of Gaussian noise.

Second, it was shown that using a lung nodule CAD system as a second reader increased the detection performance for nodules that require follow-up examinations, irrespective of the observer’s experience in chest imaging diagnosis. A figure-of-merit calculated by the JAFROC analysis represents the accuracy of the observer’s diagnosis [33]. The significant increase of the figure-of-merit when using the present system may be due to the fact that the CAD was able to comprehensively identify the lesions and that the observers correctly dismissed the FPs produced by CAD. From this result, the TP/FP rate of the CAD is at an acceptable level in cases where it is used as a second reader to reduce the omission of lesions requiring follow-up. Analysis of the effect of CAD on each type of nodule showed that it is particularly effective in increasing the number of ground-glass and small nodules detected. Ground-glass nodules may be atypical adenomatous hyperplasia, as well as adenocarcinoma in situ or another form of lung adenocarcinoma, and if they are discovered early, their prognosis is extremely good [34, 35]. If another primary lesion is present, small solid nodules may be metastatic tumors, and their presence affects staging and treatment methods. Lung nodule CAD use in actual clinical practice will improve the detection performance of these lesions, which would be of major benefit to patients.

In the group of readers who had little experience with chest image interpretation, sensitivity for nodules located in lower lobes was low compared to the more experienced groups. In general, the search for lung nodules is often performed from the apex to the bottom of the lung. Inexperienced readers took a long time to interpret the image. It is possible that the sensitivity decreased in the latter half, when the concentration tended to decrease over time. Since the detection sensitivity for nodules in the lower lobes was improved by using CAD, it is possible that CAD facilitates a consistent quality of interpretation.

In the validation test, it was confirmed that the number of FPs was very high (3.1–7.2 FPs per scan). A large number of FPs may increase the radiologists’ effort to check CAD results, resulting in a decrease in operational efficiency. However, in the present study, the high FP rates may be due to the quality of the validation dataset. To enable comparisons with previous studies, evaluations were conducted using the same datasets, but it was confirmed that these include nodules that had not been annotated, mainly tiny nodules and faint ground-glass nodules. The LNDb dataset in particular contained scans for which only some nodules had been annotated, such as those with numerous nodules, a very large number of which were counted as FPs, and the CPM score was correspondingly lower than the real situation.

In the present study, in the reader performance test, it was found that checking the CAD results increased the number of nodules picked up by a mean of 1 nodule per scan. This increase in the number of nodules picked up could increase the number of follow-up investigations, thus increasing both patients’ radiation exposure and doctors’ workload in interpreting images. Further prospective studies are needed to investigate whether CAD use will change the number of cases of lung cancer discovered and the number of follow-up investigations required. To limit any increase in unnecessary investigations, it may be necessary to use an AI system that analyzes the size and characteristics of each individual nodule and estimates its malignancy [36, 37]. The combined use of such AI may also lead to a reduction in the reading time when using CAD as a 2nd reader. Future research should evaluate clinical efficacy when combined with such an AI system.

## Conclusions

An automated lung nodule detection system was developed using deep learning that is robust to imaging conditions, and using this system as a second reader increased detection performance for nodules that require follow-up examinations.

## Supplementary Information

**Additional file 1. Fig. S1**: Lung nodule CAD detection results. TPs/FPs/FNs are abbreviations for true positives/false positives/false negatives, respectively.

**Additional file 2. Fig. S2**: Group-wise mean FROC curves using criteria 1 and 2. The red lines show the FROC curves with CAD use, and the blue lines without CAD use.

**Additional file 3. Fig. S3**: Examples of lung nodules detected by the CAD system in the reader performance test. The improvement rate shows the proportion of readers who picked up the nodule with CAD use but not without CAD use.

**Additional file 4. Table S1:** Details of data used for internal/external validation test. **Table S2**: Number and proportion of FNs.

## Data Availability

The datasets generated and/or analyzed during the current study are not publicly available because the use of patient data (CT images and reports) other than by us is not approved by the patients, but they are available from the corresponding author upon reasonable request.

## Abbreviations

CT: Computed tomography
CAD: Computer-aided detection
JAFROC: Jackknife free-response receiver-operating characteristic
AI: Artificial intelligence
CNN: Convolutional neural network
FROC: Free-response receiver-operating characteristic
FPs: False positives
CPM: Competition performance metric
SD: Standard deviation
GGN: Ground-glass nodule
TPs: True positives
FNs: False negatives
