# Supplementary figures and images for: Development and performance evaluation of a deep learning lung nodule detection system
Source: BMC Med Imaging. 2022 Nov 22;22:203. doi: 10.1186/s12880-022-00938-8 (PMC9682774; doi:10.1186/s12880-022-00938-8)

TPs

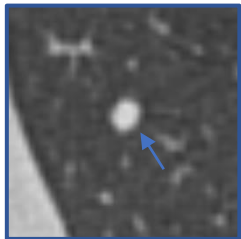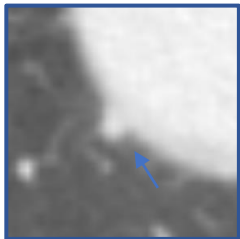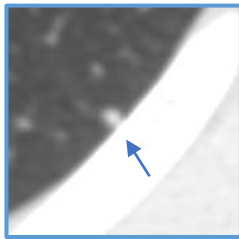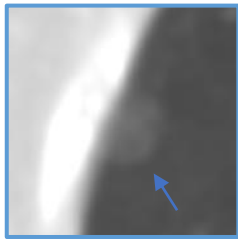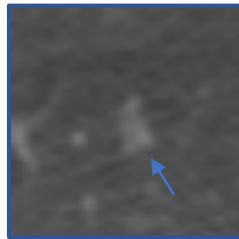

FPs

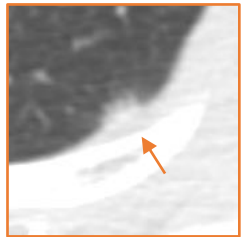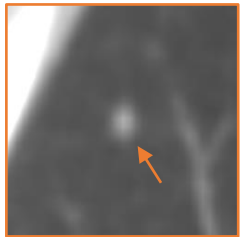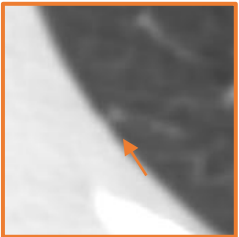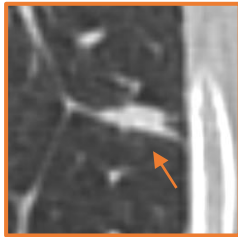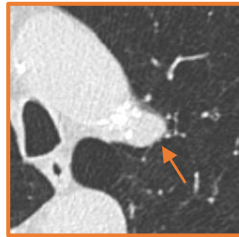

FNs

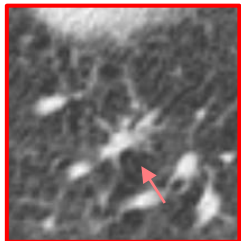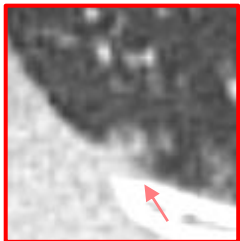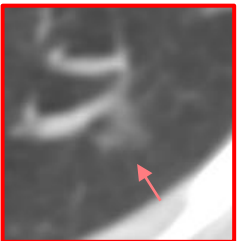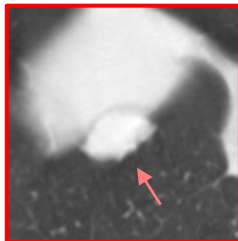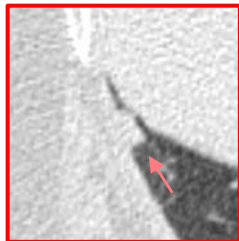

Supplement: Supplementary file 1 — Additional file 1. Fig. S1: Lung nodule CAD detection results. TPs/FPs/FNs are abbreviations for true positives/false positives/false negatives, respectively. [file 12880_2022_938_MOESM1_ESM.pdf]

Criterion 1

Criterion 2

Group 1

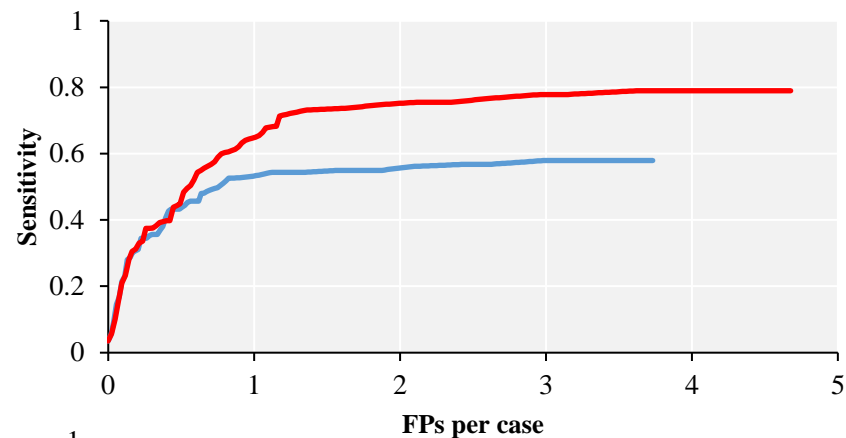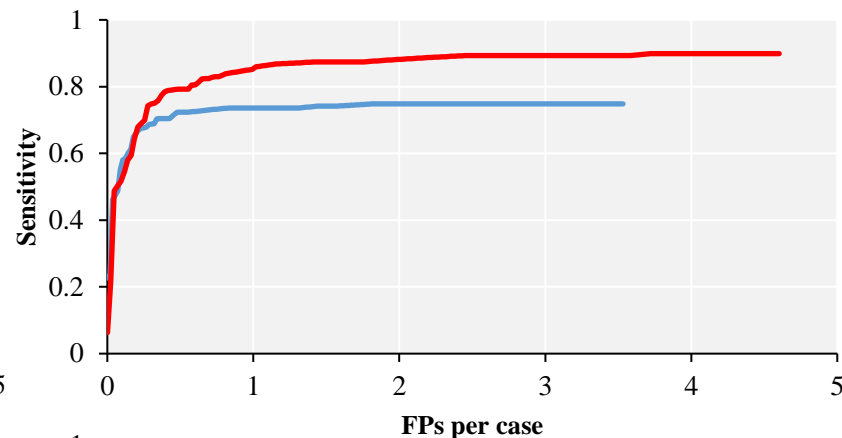

Group 2

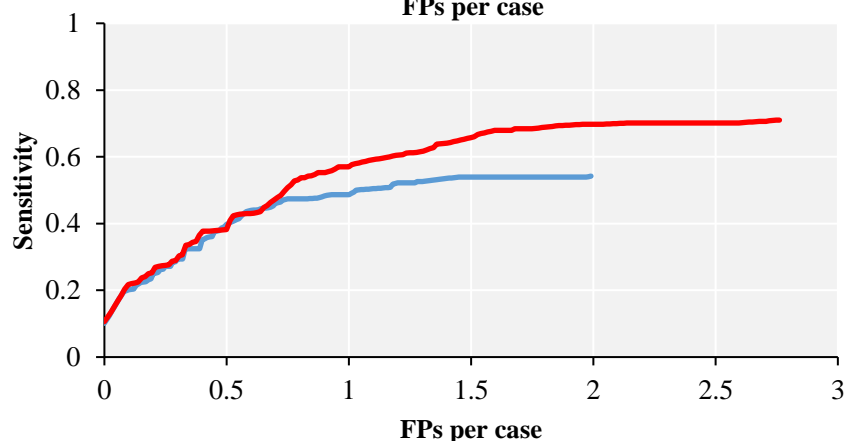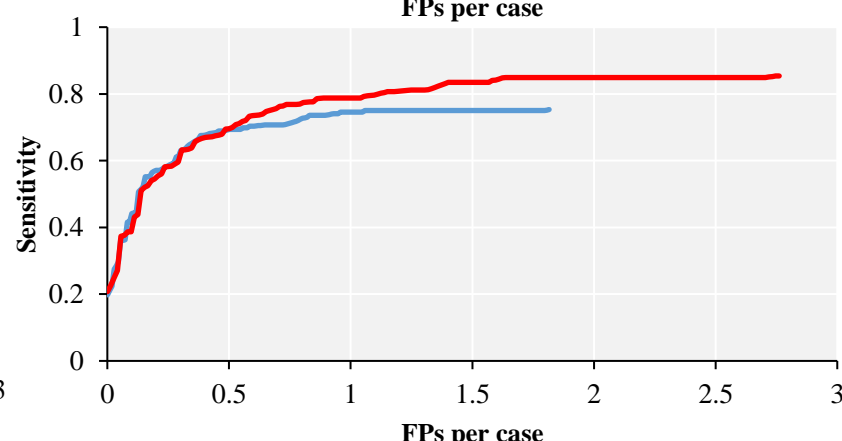

Group 3

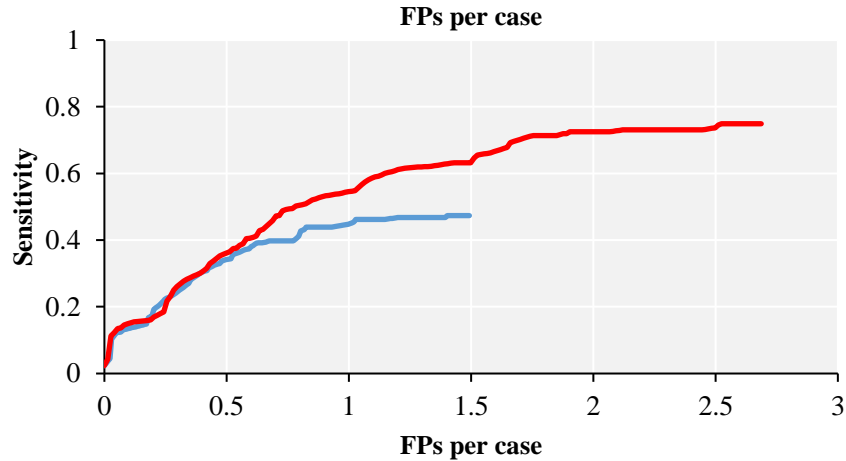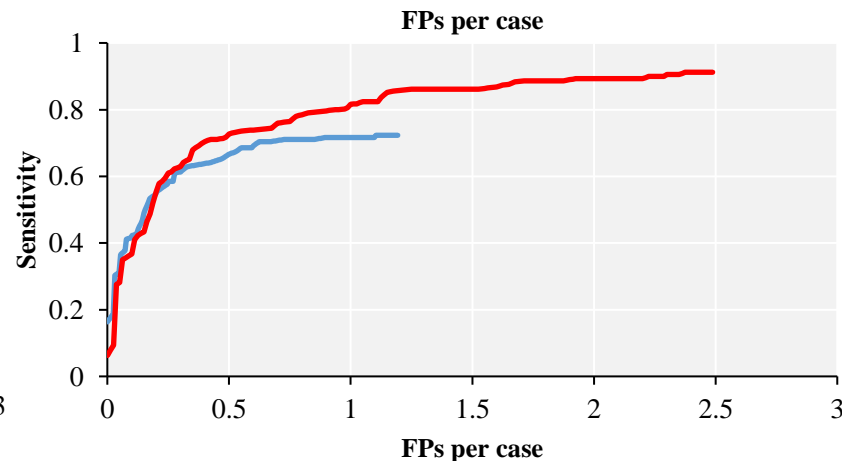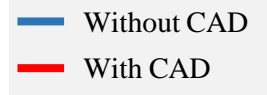

Supplement: Supplementary file 2 — Additional file 2. Fig. S2: Group-wise mean FROC curves using criteria 1 and 2. The red lines show the FROC curves with CAD use, and the blue lines without CAD use. [file 12880_2022_938_MOESM2_ESM.pdf]

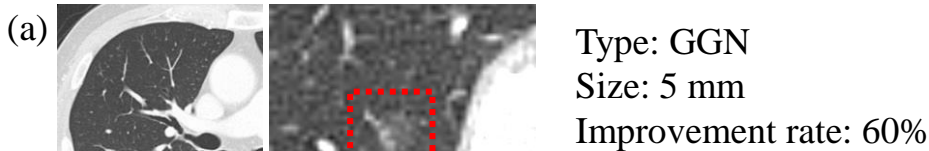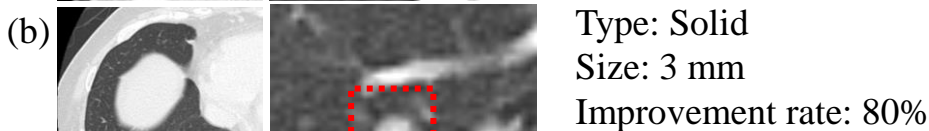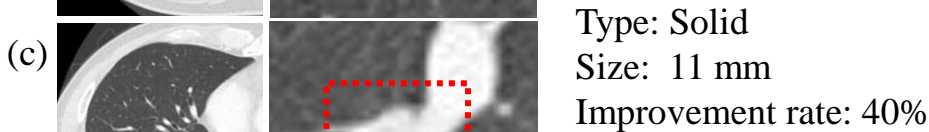

Supplement: Supplementary file 3 — Additional file 3. Fig. S3: Examples of lung nodules detected by the CAD system in the reader performance test. The improvement rate shows the proportion of readers who picked up the nodule with CAD use but not without CAD use. [file 12880_2022_938_MOESM3_ESM.pdf]
