# Supplementary material for: Development and performance evaluation of a deep learning lung nodule detection system
Source: BMC Med Imaging. 2022 Nov 22;22:203. doi: 10.1186/s12880-022-00938-8 (PMC9682774; doi:10.1186/s12880-022-00938-8)
Supplement: Supplementary file 4 — Additional file 4. Table S1: Details of data used for internal/external validation test. Table S2: Number and proportion of FNs. [file 12880_2022_938_MOESM4_ESM.docx]

Supplementary Table 1. Details of data used for internal/external validation test

| Characteristic | # | | |
| --- | --- | --- | --- |
|  | LIDC-IDRI | SPIE-AAAM | LNDb |
| Data Information | | | |
| - No. of chest CTs | 127 | 70 | 177 |
| - No. of patients |  |  |  |
| - - No. of men* | 58 | 28 | N/A |
| - - No. of women* | 59 | 42 | N/A |
| - Mean age* (y) | 58.5 ± 15.8 | 60.7 ± 13.1 | N/A |
| Slice thickness (mm) | | | |
| - - ≤1.0 | 3 | 70 | 177 |
| - - ≤2.0 | 33 | 0 | 0 |
| - - ≤3.0 | 91 | 0 | 0 |
| - Contrast |  |  |  |
| - Contrast-enhanced | 111 | 51 | 0 |
| - Non-contrast-enhanced | 16 | 19 | 177 |
| - Kernel Type |  |  |  |
| - Lung Kernel | 41 | 70 | 177 |
| - Abdomen Kernel | 86 | 0 | 0 |
| - Manufacturer |  |  |  |
| - GE Healthcare | 117 | 0 | 0 |
| - Siemens Healthineers AG | 0 | 0 | 177 |
| - Philips | 10 | 70 | 0 |
| Nodule information | | | |
| - No. of total nodules | 152 | 83 | 221 |
| - No. of nodules according to size (mm) |  |  |  |
| - - ≤10.0 | 76 | 15 | 184 |
| - - ≤20.0 | 52 | 46 | 32 |
| - - ≤ 30.0 | 17 | 16 | 5 |
| - - > 30.0 | 7 | 6 | 0 |
| - Lobular distribution |  |  |  |
| - - Right upper | 44 | 21 | 71 |
| - - Right middle | 12 | 8 | 26 |
| - - Right lower | 31 | 15 | 41 |
| - - Left upper | 36 | 24 | 48 |
| - - Left lower | 29 | 15 | 35 |
| - Internal characteristics |  |  |  |
| - - Solid | 136 | 64 | 199 |
| - - Part Solid | 7 | 15 | 7 |
| - - GGN | 9 | 4 | 15 |

*Sex/Age data have been removed from some LIDC-IDRI data, so that they are not included in the count.

Supplementary Table 2. Number and proportion of FNs.

|  | N |
| --- | --- |
| Nodule Type |  |
| - Solid | 9 |
| - Part solid | 0 |
| - Pure GGN | 4 |
| Nodule size (mm) |  |
| - ≤10 | 10 |
| - ≤ 20 | 2 |
| - ≤ 30 | 1 |
| - > 30 | 0 |
| Nodule position |  |
| - Right upper lobe | 3 |
| - Right middle lobe | 0 |
| - Right lower lobe | 4 |
| - Left upper lobe | 3 |
| - Left lower lobe | 3 |
| - Attached to parietal pleura | 3 |
| - Attached to visceral pleura | 2 |
| - Attached to interlobular fissure | 0 |
